# Annotations

**Published:** 1897-04-24

**Authors:** 


### Annotations.

### Modes of Reproduction in Plants.

The old saying, "Omne vivum ab ovo," although true enough so far as expressing the fact that all living things proceed from living things that have gone before, requires considerable modification when it is applied to various forms of vegetable life, and those not always of the "lowest" type. Thus we sometimes see plants growing one from another by a process of vegetable continuity, while the same plant may at other times propagate in a manner which may properly be called sexual, and some people have chosen to imagine that for the continuance of the species this complete cycle must be gone through. In an interesting article in *Science Progress*, however, Professor Marshall Ward, F.R.S., shows how largely mode of propagation depends on environment. Speaking of recent discoveries in regard to algæ he says: "The point is that the exact conditions which induce or govern a given biological reaction can be discovered and controlled, and so our knowledge is altered from an indefinite conviction that you must not do this, that, or the other to an Alga, or it will not grow, or multiply, or succeed generally, to the definite surety that if you want a given Alga to produce zoospores you must treat it in such and such a manner, and if you alter the treatment according to directions you can make the Alga form sexual organs, and so on." This, however, is all in accord with what we know about bacteria, which vary not only in their mode of multiplication, but even in the products of their growth, according to their pabulum and surroundings. In regard to various disease-producing bacteria, we know that they exhibit considerable inconstancy in their pathogenic activity, and it is by no means certain that this may not be related not only to variations in their surroundings, but also in their mode of reproduction.

### The Coming Despot.

Events both in the East and in the West must lead many thinkers to inquire how far freedom is a benefit to man, and whether we should not live more safely and far more pleasantly under a beneficent despotism than we do under the present rigid reign of law. The rights of property are sacred things, and not such as may lightly be disturbed, but when we see the evils which too often grow up under their ægis, one is almost inclined to cry out for an autocrat who should have power to rule according to circumstances rather than according to Act of Parliament. The condition of the tenement dwellings which have grown up in almost all great centres of population; the manner in which the land has been allowed to become literally covered with buildings in such cities as New York, London, and Liverpool; and the descriptions lately given of the insanitary conditions existing in Bombay, which have been allowed to come into being under municipal government, and under our boasted system of non-interference with the habits and customs of the natives, all tend to make people ask for a little despotism as a remedy for much municipal mismanagement. Who, then, is to be the despot? We do not think he is far to seek, nor that we shall be wrong in suggesting to those who are responsible for municipal affairs in many towns, that if they neglect their duty, the despotism under which they will fall will be one which they by no means love, that, namely, of the medical officer of health. Sometime or other, when people recognise how much disease is due to the conditions among which men live, they will arise and demand to be made healthy; they will set up a king to rule over them in the name of sanitation, and his yoke will not be easy.

### More Deaths from Anæsthetics.

We regret to have to record a further series of deaths while under the influence of anæsthetics. The *Daily Chronicle*, April 1st, reports that the Liverpool coroner had investigated the second death in that town in a week, the patient being Margaret Clements, aged 32 years, a workhouse inmate. The jury found that the death was due to excitement in anticipation of the operation. In regard to this finding, we may draw attention to the results of some investigations lately made by Dr. Leonard Hill, who finds that chloroform paralyses the splanchnic vaso-motor tone just as shock does, and that "in the condition of shock or emotional fear the compensatory mechanism for the effect of gravity is almost abolished, and chloroform may easily be the last straw to completely paralyse the circulation." It is, no doubt, in this way that an expectation of disaster has sometimes conduced to its occurrence. The *Star*, April 6th, reports the death of a printer's errand boy under chloroform, administered for the performance of an operation on the eye. He took the chloroform badly, holding his breath and exhibiting symptoms of alarm. His breath suddenly ceased, and he was found to be dead. The *Derbyshire Courier*, April 10th, reports an inquest held at the hospital on a boy, one year and seven months old, who died under chloroform. The operation was the opening of an abscess in the arm. The *South London Mail*, April 10th, reports an inquest on the body of a woman, aged 35, at the Camberwell Infirmary. On admission she was suffering from hernia. Ether was administered, but the operation had scarcely been commenced when it was found that she was so ill that the anæsthetic had to be discontinued. Tracheotomy was performed, but the patient died. The cause of death was asphyxia from inspiring vomited matter. The *Glasgow Daily Record*, April 10th, records the death of a lady under chloroform administered for tooth extraction.

### The Port of London.

The report of Dr. Collingridge, Medical Officer of Health to the Port of London, is an interesting document and a record of good work. It is a matter of no small labour to administer sanitary law on a great river like the Thames, and Dr. Collingridge is to be congratulated on the manner in which he has carried out his multifarious duties, including as they did the visiting and inspection of 14,422 vessels in the course of half a year. It must not be imagined that Dr. Collingridge confines his attention to protecting London from the introduction of disease; he also watches over the health conditions under which the sailors live who conduct the traffic, and in regard to this he draws attention to various sanitary defects in their accommodation. A short account is given of the two cases of plague which arrived in the port last September; and a record is also given of the large amount of food stuffs which were found to be unfit for food consumption and were therefore destroyed. Altogether an interesting and good report.

